# Changes in health-related quality of life over the first three months of medical marijuana use

**DOI:** 10.1186/s42238-024-00245-9

**Published:** 2024-09-11

**Authors:** Michelle R. Lent, Thomas R. McCalmont, Megan M. Short, Karen L. Dugosh

**Affiliations:** 1 Department of Clinical Psychology, Philadelphia College of Osteopathic Medicine, 4170 City Avenue, Rowland Hall Philadelphia, Philadelphia, PA 19131 USA; 2 Research & Evaluation Group, Public Health Management Corporation, Philadelphia, PA USA

**Keywords:** Health-related quality of life, Medical marijuana, Short Form-36

## Abstract

**Background:**

The psychosocial impact of medical marijuana use is not yet known. This study evaluated short-term changes in health-related quality of life (HRQoL) over the first three months of medical marijuana use.

**Methods:**

This prospective, observational, longitudinal study followed adults newly recommended for medical marijuana by a physician for any of the more than 20 qualifying medical conditions in Pennsylvania. Participants (*N* = 438) provided their clinical status and demographic information, and completed semi-structured interviews prior to medical marijuana initiation (baseline) and at three months. HRQoL was assessed by the Short Form-36 (SF-36). Paired-samples *t*-tests evaluated changes in HRQoL over time.

**Results:**

Participants (*M* age = 46.4 years [15.6]; 66.4% female) were mostly commonly referred for medical marijuana to treat anxiety disorders (61.9%) or severe chronic or intractable pain (53.6%). Participants reported rapid and significant improvements in all of the domains of HRQoL from baseline to three months after initiating medical marijuana use (physical functioning, role limitations due to physical health problems, emotional well-being, role limitations due to emotional problems, bodily pain, social functioning, energy/fatigue and general health, *P* < .001 for all). Age was negatively predictive of level of improvement over time for the physical functioning (*P* < .0001), role limitations due to physical health problems (*P* < .001), and pain (*P* < .0001) domains after controlling for baseline, with older participants displaying less improvement than younger participants.

**Conclusions:**

Gains were observed in all HRQoL domains assessed after three months of medical marijuana use. In several domains, age was a significant predictor of degree of improvement.

Despite the increasing utilization of marijuana for medical purposes [1, 2], the effects of medical marijuana use on the health-related quality of life (HRQoL) of individuals who begin using cannabis-based products for medical reasons are largely unknown. According to the Centers for Disease Control and Prevention [3], HRQoL is a multidimensional construct that reflects “physical wellbeing, social and emotional wellbeing, and the impact of social determinants of health” (p.1). While the assessment of HRQoL provides valuable information regarding the wellbeing of individuals, including those living with chronic medical conditions, studies suggest HRQoL has prognostic value in regards to clinical outcomes such as mortality risk in older adults and in individuals with cancer diagnoses [4, 5].

Ongoing monitoring of HRQoL in individuals using medical marijuana to treat various mental and physical health conditions has the potential to capture changes in both the burden of the referring condition and the impact of medical marijuana on the wellbeing and functioning of users as treatment progresses. Several studies have demonstrated HRQoL scores in individuals initiating medical marijuana treatment to be comparable, or in some domains lower, than those observed in other studies of individuals with chronic health conditions [6, 7]. Furthermore, a number of studies provide preliminary evidence that medical marijuana treatment may enhance patients’ HRQoL [8–10]. For example, a prospective three-month study of medical marijuana users in the UK reported improvements in the pain/discomfort and anxiety/depression domains of HRQoL [9] and a retrospective case series of medical marijuana users in Australia reported similar findings in the domains of pain and mental health [11]. In Florida, middle aged and older patients using medical marijuana for pain reported higher HRQoL three months after baseline [10]. However, a review of studies evaluating HRQoL and medical marijuana found the existing literature to be inconclusive for conditions other than pain and called for more rigorous study designs and longer study durations in future investigations [12–15]. Studies employing prospective designs and larger samples, utilizing well-established QoL measures, and recruiting patients when they first begin medical marijuana use, are needed to clarify the relationship between HRQoL and medical marijuana use.

The current prospective, longitudinal study evaluated changes in eight domains of HRQoL over the first three months of medical marijuana treatment in a large sample of adults initiating medical marijuana treatment for the first time in Pennsylvania (PA) for any of the more than 20 approved health conditions, which include mental health disorders (e.g., anxiety disorders, posttraumatic stress disorder) and physical health disorders (e.g., severe chronic or intractable pain, neurodegenerative diseases, inflammatory bowel disease, intractable seizures). This study included only new medical marijuana users to most effectively capture changes in HRQoL with the initiation of medical marijuana use, and utilized a well-validated measure of HRQoL, the Short Form-36 (SF-36) [16, 17]. We hypothesized that adults would report significant improvements in all aspects of HRQoL from baseline to three months after initiating medical marijuana use. In addition, we examined potential predictors of change in HRQoL during this same time period.

## Methods

Individuals aged 18 years or older were eligible to participate in the study if they had obtained a recommendation from a PA physician for medical marijuana to treat any qualifying medical condition, had a valid state-issued medical marijuana card, and were naïve to medical marijuana. Individuals were excluded from participation if they were unable to provide informed consent, reported active heavy recreational marijuana use (use on most days for the past month), or were not English speaking. Recruitment occurred at four dispensaries in the greater Harrisburg and Pittsburgh, PA areas.

Potentially eligible individuals were introduced to onsite study research staff by the dispensary pharmacists after they presented for their initial medical marijuana consultation. New medical marijuana patients at the dispensary sites where recruitment took place meet with a pharmacist prior to their initial medical marijuana purchase. Interested individuals completed informed consent, granted research staff access their dispensary medical record, and completed the structured baseline interview including the HRQoL measure within one week of consent. Participants consented to be followed over the first year of medical marijuana use and to return for study visits at 3, 6, 9 and 12-months post-enrollment. Follow-up study assessments were completed either in person during dispensary visits to purchase products or via phone. Participants’ medical marijuana purchases from dispensaries were collected over the study year. In general, the baseline appointment, including the informed consent process, took approximately 60–90 min to complete. Participants were remunerated $25 USD for each study visit completed. Baseline HRQoL data were previously reported [7].

Study data were collected and managed using REDCap (Research Electronic Data Capture) hosted at the lead institution. REDCap is a secure, web-based software platform designed to support data capture for research studies.[18] The Philadelphia College of Osteopathic Medicine Institutional Review Board (#H17-060) approved the research protocol and provided ongoing ethical oversight of the study. The dataset analyzed for the current study is available in the DigitalCommons repository, https://digitalcommons.pcom.edu/.

### Measures

#### Demographic and Medical Information

Participants reported their age, identified gender, marital status, race, ethnicity, completed education level, socioeconomic status (e.g., household income), current medical diagnoses, and the medical reason(s) for their medical marijuana recommendation. Current (past 90 days) and lifetime use of recreational marijuana was collected using the Addiction Severity Index (ASI).[19].

#### Health-Related Quality of Life (HRQoL)

The Short-Form—36 (SF-36) is a 36-item structured clinical assessment evaluating physical and mental health functioning, with most questions referring to the past four weeks.[16, 17] The SF-36 measures several areas of HRQoL: physical functioning, role limitations due to physical health problems, emotional well-being, role limitations due to emotional problems, social functioning, energy/fatigue, bodily pain, and general health. Scores for each scale range from 0 (poorest) to 100 (highest). The SF-36 was administered at baseline (prior to the start of medical marijuana use) and again three months after starting medical marijuana (± 2 weeks). Lower scores are indicative of poorer functioning.

### Statistical approach

Descriptive statistics (mean, standard deviations and frequencies) were generated to characterize the overall sample at study entry. A series of paired t-tests were performed to evaluate the extent to which HRQoL subscale scores differed significantly from baseline to month three for each domain. Finally, a series of linear regression analyses were used to examine the extent to which participant-level characteristics at baseline (i.e., age, race, gender identity [= female in analyses], chronic pain as a referring condition, anxiety as a referring condition) were associated with the degree of change observed at month three for each subscale of the SF—36 (i.e., month three scale score – baseline scale score) after controlling for the corresponding baseline score. For each subscale, t-test and regression analyses included all individuals with non-missing data at baseline and the three-month follow-up assessments. Effect sizes (Cohen’s *d*) were calculated to estimate clinically significant change in each HRQoL domain. Significance levels were set at *P* < 0.05.

## Results

From September 2020 – June 2023, 1,314 new medical marijuana patients were approached regarding the study and 452 (34.4%) enrolled and completed the baseline assessment. Of the 452 enrolled, 14 participants withdrew prior to the three-month assessment (*N* = 438). Most individuals who declined participation reported time constraints and a desire to initiate medical marijuana treatment immediately (vs. after completion of the baseline assessment) as the primary reason they did not enroll in the study. In total, 399 participants completed the three-month follow-up assessment (91% follow-up rate) and were included in the longitudinal HRQoL analyses. At the time of these analyses, the study was ongoing and data collection continued for one-year post-enrollment.

### Participant characteristics

Participants (*N* = 438) were a mean age of 46.4 years (*SD* = 15.6) and predominantly identified as White (94.5%) and female (66.4%). The majority of participants were referred for medical marijuana for the treatment of chronic intractable pain (53.6%) and/or an anxiety disorder (61.9%). Sample characteristics are presented in Table 1.

**Table 1:** Sample characteristics of first-time medical marijuana users in Pennsylvania (*N* = 438)

	*Mean (SD)*	*n* (%)
Age (years)	46.4 (15.6)	
Gender		
Male		142 (32.6)
Female		289 (66.4)
Transgender, Non-binary, Not Specified		7 (1.0)
Race		
White		414 (94.5)
Black		16 (3.7)
Other		8 (1.8)
Latino/a/x Ethnicity		12 (2.7)
Marital Status		
Married or Domestic Partnership		244 (55.7)
Single (Never Married)		108 (24.6)
Separated or Divorced		69 (15.8)
Widowed or Unknown		17 (3.9)
Highest Degree Completed		
High School Diploma or GED		101 (23.1)
Associate’s Degree or Trade/Technical		70 (16.0)
Bachelor’s Degree		106 (24.3)
Graduate Degree		59 (13.5)
Other		102 (23.3)
Most Common Referring Conditions for Medical Marijuana*		
Chronic or intractable pain		235 (53.7)
Anxiety disorder		271 (61.9)
Post-Traumatic Stress Disorder		50 (11.4)
Neuropathies		27 (6.2)
Cancer		25 (5.7)
Irritable Bowel Syndrome		17 (3.9)
Multiple Sclerosis		11 (2.5)
Glaucoma		6 (1.4)
Recreational Marijuana Use		
Any use, past 90 days (yes)		107 (25.4)
Any use, lifetime (yes)		222 (52.7)
Medical Marijuana Product Type Purchased Over 3 Months**		
Oral		237 (56.0)
Vape/Inhaled		256 (60.5)
Concentrate/Extract		77 (18.2)
Topical		111 (26.2)

### Changes in HRQoL scale scores from baseline to month 3

**Fig. 1:**
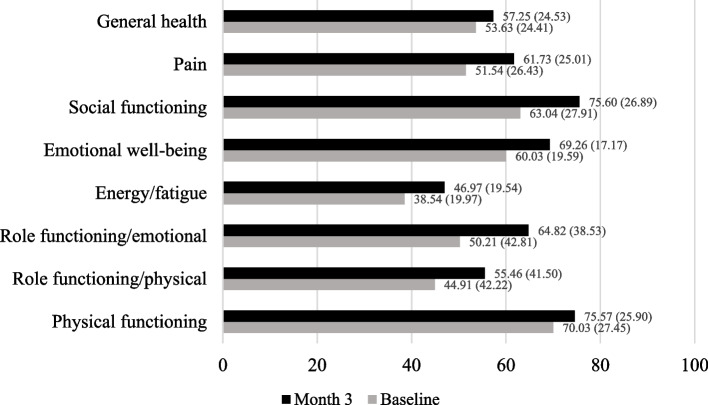
Changes* in Health-Related Quality of Life (HRQoL) Over Three Months of Medical Marijuana Use (*N* = 399). * Paired-samples t-tests, all scales significant at <.001. HRQoL measured by the Short Form-36

Participants reported significant improvements across all domains of HRQoL after the first three months of medical marijuana use (see Fig. 1, *P*'s < 0.001 for all domains). Specifically, participants endorsed gains from baseline to month three in physical functioning (*t*[395] = -5.23, + 6.5%, *d* = 0.26), role limitations due to physical health problems (*t*[397] = -5.59, + 23.5%, *d* = 0.28), role limitations due to emotional problems (*t*[396] = -7.47, + 29.1%, *d* = 0.37), energy/fatigue (*t*[397] = -9.31, 18.8%, *d* = 0.47), emotional well-being (*t*[397] = -11.25, + 15.4%, *d* = 0.56), social functioning (*t*[396] = -9.08, + 25.9%, *d* = 0.46), bodily pain (*t*[396] = -9.54, + 19.8%, *d* = 0.48), and general health (*t*[394] = -4.81, + 6.7%, *d* = 0.24).

### Predictors of HRQoL change at month three

Results from the series of regression analyses predicting the three-month change score are presented in Table 2. Baseline score was a significant predictor of degree of change across each of the SF-36 domains (all *P*’s < 0.0001); participants with higher baseline HRQoL scores displayed lower levels of change than those with lower baseline HRQoL scores within each domain. In addition, age significantly predicted the observed degree of change for the physical functioning (*t(*Bruce et al. [7] June [7]) = -4.70, *P* < 0.001), role limitations due to physical health problems, (*t(*Bruce et al. [7] June [7]) = -3.71, *P* < 0.001), and bodily pain (*t(*Bruce et al. [7] June [7]) = -4.46, *P* < 0.0001) domains. Specifically, age was negatively associated with the degree of observed change with older participants displaying less improvement from baseline to the three-month follow-up than younger participants. Gender, anxiety as a referring condition, and chronic pain as a referring condition did not reach statistical significance in any of the models.

**Table 2:** Regression analyses predicting change score at month three for each SF-36 domain

Domain	Variable	Beta	SE	t	R^2^ for model
Physical functioning (*n* = 393)	(Intercept)****	41.66	4.68	8.90	0.22
	Baseline score****	-0.33	0.03	-10.03	
	Female gender	-1.00	1.66	-0.60	
	Age****	-0.27	0.06	-4.70	
	Anxiety	0.30	1.85	0.16	
	Chronic pain	-1.63	1.81	-0.90	
Role limitations due to physical health (*n* = 395)	(Intercept)****	58.89	7.60	7.75	0.26
	Baseline score****	-0.49	0.04	-11.29	
	Female gender	-6.71	3.53	-1.90	
	Age***	-0.43	0.12	-3.71	
	Anxiety	1.45	3.92	0.37	
	Chronic pain	-5.32	3.82	-1.40	
Role limitations due to emotional problems (*n* = 394)	(Intercept)****	38.65	7.27	5.31	0.30
	Baseline score****	-0.50	0.04	-11.92	
	Female gender	1.09	3.53	0.31	
	Age	0.11	0.12	0.94	
	Anxiety	-2.65	3.94	-0.67	
	Chronic pain	-5.41	3.72	-1.46	
Energy/fatigue (*n* = 395)	(Intercept)****	27.90	3.80	7.34	0.23
	Baseline score****	-0.44	0.04	-10.61	
	Female gender	-2.27	1.73	-1.31	
	Age	-0.02	0.06	-0.42	
	Anxiety	0.79	1.88	0.42	
	Chronic pain	-1.01	1.81	-0.56	
Emotional well-being (*n* = 395)	(Intercept)****	37.06	3.72	9.97	0.31
	Baseline score****	-0.46	0.04	-12.18	
	Female gender	0.98	1.48	0.66	
	Age	-0.01	0.05	-0.16	
	Anxiety	-0.42	1.65	-0.26	
	Chronic pain	-0.10	1.55	-0.06	
Social functioning (*n* = 394)	(Intercept)****	50.18	5.77	8.69	0.29
	Baseline score****	-0.53	0.04	-12.19	
	Female gender	-3.65	2.53	-1.44	
	Age	-0.05	0.08	-0.56	
	Anxiety	1.98	2.77	0.71	
	Chronic pain	-2.08	2.67	-0.78	
Pain (*n* = 394)	(Intercept)****	45.39	4.99	9.10	0.27
	Baseline score****	-0.44	0.04	-10.69	
	Female gender	0.79	2.00	0.40	
	Age****	-0.30	0.07	-4.46	
	Anxiety	1.42	2.18	0.65	
	Chronic pain	-0.70	2.25	-0.31	
General health (*n* = 392)	(Intercept)****	15.09	3.43	4.40	0.12
	Baseline score****	-0.20	0.03	-6.82	
	Female gender	1.64	1.51	1.08	
	Age	-0.02	0.05	-0.34	
	Anxiety	0.79673	1.66	0.48	
	Chronic pain	-2.44937	1.61	-1.52	

Note: *p < .05, **p < .01, ***p < .001, ****p < *.0001*

## Discussion

As hypothesized, new medical marijuana users experienced improvements across all domains of HRQoL over the first three months of medical marijuana use for any of the more than 20 qualifying medical conditions for use in PA. Notably, participants endorsed greater than 20% increases in ratings of their role limitations due to physical health problems and emotional problems, and in social functioning after three months of medical marijuana use. The physical health and emotional limitations subscales of the SF-36 capture time spent, amount accomplished, or difficulties encountered when performing work or other activities due to physical health or emotional problems over the past four weeks, while the social functioning subscale assesses the extent to which physical or emotional problems have interfered with normal social activities.[16] We believe that these HRQoL gains represent clinically meaningful change in our participants. According to Wyrwich and colleagues, “clinically significant change in QoL is a difference score that is large enough to have an implication for the patient’s treatment or care” (p. 286).[20] While our study did not ask participants to define this threshold, the moderate effect sizes found in several domains including energy/fatigue, emotional well-being, social functioning, and bodily pain suggest that meaningful change occurred. Further, HRQoL directly relates to clinical outcomes. In individuals with chronic pain conditions, greater severity of pain is associated with greater impairments in HRQoL [21]; however, HRQoL improves as pain levels decline with treatment.[22].

Notably, the changes in HRQoL seen in our study were comparable to HRQoL gains in others evaluating different treatment modalities for the two most common referring conditions, anxiety and chronic pain. A study of HRQoL after 13 weeks of oral analgesic use for chronic knee pain found improvements in bodily pain scores on the SF-36 that were similar to the changes in bodily pain scores in our study after 12 weeks of medical marijuana use.[23] HRQoL has also been evaluated in individuals with generalized anxiety disorder (GAD). A double-blind, placebo-controlled study evaluating HRQoL following treatment with the prescription medication vortioxetine for GAD also found similar gains in social functioning scores after eight weeks as we found after 12 weeks of medical marijuana use.[24] Future studies that employ randomized controlled designs are needed to better understand the effectiveness of medical marijuana on HRQoL compared to other treatments for various conditions.

How the use of medical marijuana may relate to the observed gains in HRQoL, particularly in the domains that increased most, requires further study. For example, levels of energy increased in medical marijuana users as reported in the energy/fatigue subscale; future studies may benefit from examining potential mediators of this effect such as sleep quality and duration. It is also plausible that levels of bodily pain could mediate the gains observed at three months in physical and social functioning, especially given the high number of participants referred for medical marijuana to treat chronic pain. Additionally, the observed social functioning gains may be particularly relevant to individuals referred for medical marijuana for the treatment of anxiety disorders, as the symptoms associated with many anxiety disorders can substantially interfere with interpersonal functioning. The HRQoL gains in our study are similar to those reported in a study of patients with chronic pain [10] and in a smaller study of UK patients over three months of medical marijuana use.[9] Notably, referring condition (i.e., anxiety, chronic pain) was not related to the degree of improvement in HRQoL for any of the domains examined. Older participants in our study, however, reported less robust gains in their physical functioning and pain than younger participants, which may reflect the changes in functioning that normally coincide with older age, or may indicate a possible limitation of medical marijuana use in older adults. Additional studies are needed to clarify this finding.

This study has multiple strengths. To our knowledge, this study is one of the largest longitudinal studies of quality of life in individuals using medical marijuana in the US. This study utilized a well-established and widely used measure of HRQoL in both research and clinical domains, and had a high follow-up rates at month three (91%). The study also had several limitations. Our sample identified as predominantly White and female, limiting the generalizability of our findings to other groups. The reasons why this particular demographic is overrepresented are unclear, but may relate to the higher prevalence of anxiety disorders [25] and chronic pain [26] in women (the two most common referring conditions in this study), the greater likelihood of women to utilize complementary and alternative medicine compared to men, or fears of potentially negative outcomes in racial and ethnic minorities despite the legalization of cannabis use for medicinal purposes.[27] The observational study design did not allow for the attribution of causality in regards to the observed changes in HRQoL and the initiation of medical marijuana use; the current Schedule I designation of products containing tetrahydrocannabinol (THC) in the US limits the utilization of more rigorous study designs.

Individuals seeking medical marijuana for the first time may be experiencing particularly acute, intense or refractory medical conditions that more adversely impact their HRQoL compared to those seeking more evidence-based behavioral or prescription treatment options. It is also important to note that while individuals who enrolled in this study were naïve to medical marijuana use, 25% reported recent recreational use. The rate of recent recreational marijuana use was higher than rates of marijuana use reported in the general U.S. population with medical conditions [28] and could therefore limit the generalizability of our findings. Further, the high number of participants reporting previous experience with marijuana allows for the potential for both expectancy biases and placebo effects surrounding the use of marijuana for medical purposes. Beyond the reasons for declining study enrollment, no additional information was collected regarding the individuals who declined to participate in this study. It is possible that these individuals were different than enrolled participants in meaningful ways and future studies may benefit from obtaining descriptive information on those who decline to participate. While the majority of participants who did not complete three-month assessments were lost to follow-up (> 80%) and therefore the reasons for drop out remain unknown, several participants reported their reasons for dropping to be low remuneration and a high burden of time. Further, the present analyses did not evaluate potentially adverse psychosocial outcomes of medical marijuana use, though Arkell and colleagues found use to be associated with a low risk of serious adverse events.[11] Future studies could evaluate the adverse or unintended consequences of use or the reasons for initiating use, and may benefit from following participants for longer durations. The present study represents interim analyses and future analyses of this ongoing study will help to determine whether the observed gains in HRQoL at three months are sustained over the first year of medical marijuana use.

In conclusion, the use of medical marijuana for three months was associated with improvements in physical, social, emotional and pain-related HRQoL. Ongoing surveillance of HRQoL in individuals with physical and mental health conditions can help to treat the “whole person” and to capture any collateral impact of selected therapeutic approaches as treatment initiates and progresses. Results from this study can help patients, their caregivers, and their providers to make more informed and evidence-based decisions on whether to incorporate medical marijuana into their treatment regimens.

## Data Availability

Study data and a data dictionary are available at https://digitalcommons.pcom.edu/.

## Abbreviation

HRQoL: Health-related quality of life
